# Effectiveness and safety of stem cell therapy for diabetic foot: a meta-analysis update

**DOI:** 10.1186/s13287-022-03110-9

**Published:** 2022-08-13

**Authors:** Yuming Sun, Jinhong Zhao, Lifang Zhang, Zhexuan Li, Shaorong Lei

**Affiliations:** 1 Department of Plastic and Cosmetic Surgery, Xiangya Hospital, Central South University, 87 Xiangya Road, Changsha, 410008 Hunan Province China; 2 School of Health Policy and Management, Chinese Academy of Medical Sciences & Peking Union Medical College, Beijing, 100730 China

**Keywords:** Diabetic foot, Stem cell, Meta-analysis

## Abstract

**Background:**

Diabetic foot (DF) is one of the most common and serious complications of diabetes mellitus (DM), which brings great psychological and economic pressure to patients. This study aimed to evaluate the efficacy of stem cells in the treatment of diabetic foot.

**Methods:**

All relevant studies in Cochrane, Embase, PubMed, Web of Science, China National Knowledge Infrastructure, and WanFang databases were systematically searched for meta-analysis. The outcomes consisted of ulcer or wound healing rate, amputation rate, new vessels, ankle–brachial index (ABI), transcutaneous oxygen pressure (TcPO2), pain-free walking distance, and rest pain score. Dichotomous outcomes were described as risk ratios (RR) with 95% confidence intervals (CIs), while continuous data were presented as standardized mean differences (SMDs) with 95% CIs. Statistical analysis was performed with RevMan 5.3 software.

**Results:**

A total of 14 studies with 683 participants were included in the meta-analysis. Meta-analysis showed that stem cell therapy was more effective than conventional therapy in terms of ulcer or wound healing rate [OR = 8.20 (5.33, 12.62)], improvement in lower extremity ischemia(new vessels) [OR = 16.48 (2.88, 94.18)], ABI [MD = 0.13 (0.04, 0.08)], TcO2[MD = 4.23 (1.82, 6.65)], pain-free walking distance [MD = 220.79 (82.10, 359.48)], and rest pain score [MD = − 1.94 (− 2.50, − 1.39)], while the amputation rate was significantly decreased [OR = 0.19 (0.10, 0.36)].

**Conclusions:**

The meta-analysis of the current studies has shown that stem cells are significantly more effective than traditional methods in the treatment of diabetic foot and can improve the quality of life of patients after treatment. Future studies should conduct large-scale, randomized, double-blind, placebo-controlled, multicenter trials with high-quality long-term follow-up to demonstrate the most effective cell types and therapeutic parameters for the treatment of diabetic foot.

**Supplementary Information:**

The online version contains supplementary material available at 10.1186/s13287-022-03110-9.

## Introduction

Diabetes mellitus is one of the most common chronic metabolic disorders, with 536.6 million people living with diabetes worldwide in 2021, and it is estimated that this number will rise to 783.2 million in 2045 [1]. According to International Diabetes Federation (IDF) estimates, around 50% of people with diabetes are unaware of their condition [2, 3]. DM brings a heavy psychological and financial burden to patients, and diabetes-related health expenditures were estimated at 966 billion USD in 2021 [1]. Diabetic foot is one of the most common and serious complications, and up to 20% of diabetic patients require hospitalization for DF [4]. Neuropathy and ischemia are the main pathological changes of the DF, leading to ulceration and, in severe cases, increased risk of amputation or death [5, 6].

Current standard regimens for DF treatment include metabolic control and comorbidity treatment, infection treatment, tissue perfusion restoration, local ulcer debridement care, wound dressings, pressure off-loading, vascular surgery, and other procedures [7–9]. Chronic diabetic foot is characterized by a damaged regeneration process. Most of the current treatment methods for DF target a single factor of wound healing, while stem cell treatment of DF can correct the factors leading to long-term wound healing through various mechanisms [10, 11]. The mechanisms include promoting collagen deposition, promoting new blood vessel formation, and improving lower extremity ischemia and inflammation [12]. In recent years, a large number of clinical trials have demonstrated the potential of stem cells in the treatment of DF [11].

Although several meta-analyses have reported the efficacy of stem cells in the treatment of diabetic ulcers, their sample size is small, and the outcome indicators are relatively single [13–16]. To more fully demonstrate the accuracy of the conclusions, it is necessary to include more studies to update the meta-analysis. Our study included more clinical trials and analyzed multiple indicators of DF prognosis.

## Material and methods

### Information sources and search strategy

We systematically searched Cochrane, Embase, PubMed, Web of Science, China National Knowledge Infrastructure, and WanFang databases for all related literature works. The final search was updated on April 10, 2022, using the terms (("stem cell*"[Title/Abstract] OR "bone marrow"[Title/Abstract] OR "progenitor cell*"[Title/Abstract] OR "lipoaspirate cell*"[Title/Abstract] OR "mononuclear cell*"[Title/Abstract]) AND ("diabetic*"[Title/Abstract] OR "diabetic*"[Title/Abstract])) AND ("wound"[Title/Abstract] OR "ulcer"[Title/Abstract] OR "foot"[Title/Abstract] OR "ischemia"[Title/Abstract] OR "ischaemia"[Title/Abstract]. The publication language was restricted to Chinese and English. The original and review articles were manually identified, and the references that met the requirements were included in this study.

### Eligibility criteria

The studies were eligible if they adhered to the following criteria: (1) publication in English or Chinese language, (2) only controlled trials involving human subjects, (3) recruit patients with diabetic foot and divide them into a local treatment group using stem cells or a control group (with no treatment or placebo), and (4) report of one or more outcomes regarding the healing of the ulcers or wound, amputation, new vessels, ABI, TcPO2, pain-free walking distance, and rest pain.

### Study selection and data extraction

Two authors (JH Z and LF Z) independently performed the literature search, data extraction, and quality assessment process according to the inclusion criteria. All disputes between the two authors were resolved by discussion with the third author (ZX L). Studies selection is based on title and abstract, with full text reviewed as necessary. The following data were extracted from studies that met the inclusion criteria: countries of the studies, characteristics of participants, year of publication, study design, intervention and regimen details, clinical endpoints, and follow-up period. We extracted ulcer or wound healing rate, amputation rate, new vessels, ABI, TcPO2, pain-free walking distance, and rest pain data to evaluate the effect of stem cell therapy on diabetic foot. Safety assessment included any adverse events (AEs) during stem cell therapy.

### Quality assessment

The methodology of included systematic reviews was evaluated using the Cochrane Risk of Bias tool; evaluation indicators include: (1) whether the method of random sequence generation is correct (selection bias), (2) whether to achieve allocation concealment (selection bias), (3) whether to use blind methods for participants and implementers (performance bias), (4) whether to use blindness for outcome measurers (detection bias), (5) whether the result data are complete (attrition bias), (6) whether to report selectively (reporting bias), and (7) other bias. RevMan version 5.3 to generate the risk of bias tables. Study quality was assessed by one reviewer and checked by another. Any differences were resolved through discussion.

### Data synthesis and analysis

A meta-analysis of only controlled trials including stem cell therapy regimens for the diabetic foot was performed. For these dichotomous results, we used estimated odds ratios (ORs) and 95% confidence intervals, while continuous data were presented as standardized mean differences (SMDs) with 95% CIs. *I*^2^ was used to assess heterogeneity among included studies, and if *I*^2^ ≤ 50%, we used a fixed-effects model. If *I*^*2*^ > 50%, it is considered that there is statistically significant heterogeneity [17], and we choose a random effect model to combine the results to reduce the influence of heterogeneity. Sensitivity analyses were performed to examine studies that contributed to heterogeneity. Subgroup analyses were performed to assess the impact of certain study characteristics on outcomes with high heterogeneity. Funnel plot asymmetry was measured to assess publication bias. Probability values of < 0.05 were considered statistically significant. All statistical analyses were conducted using RevMan Software (Version 5.3. Copenhagen: The Nordic Cochrane Centre, the Cochrane Collaboration, 2014).

## Results

### Search results

**Fig. 1:**
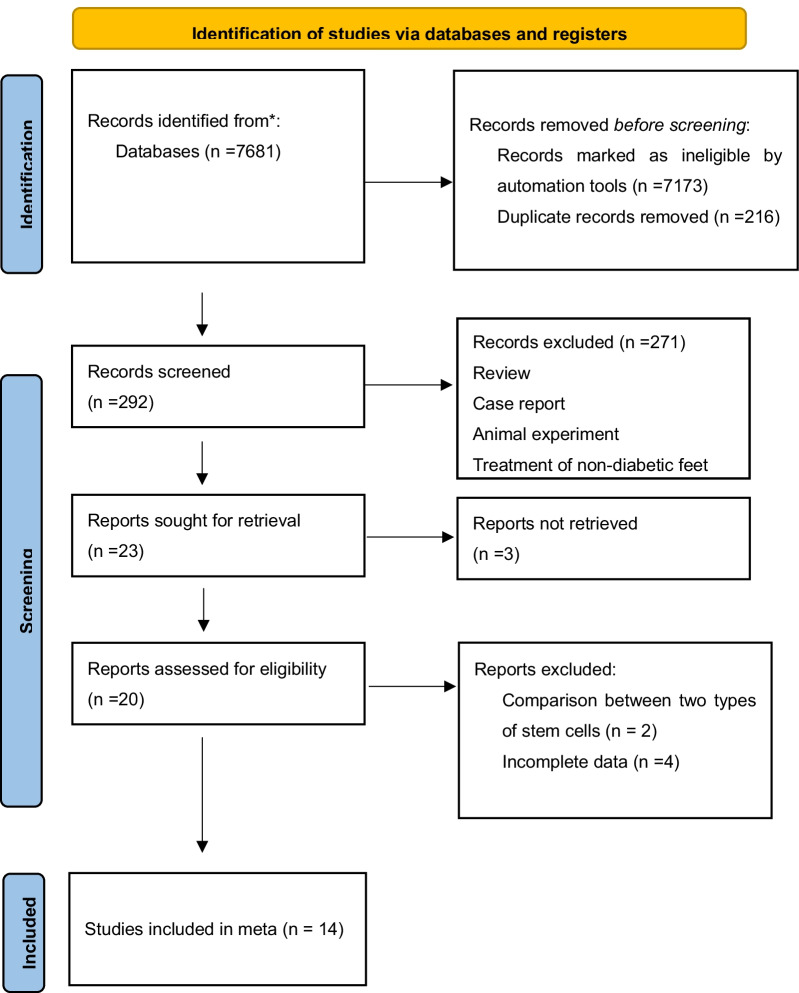
Flow chart of study selection in the meta-analysis

The latest version of the PRISMA flowchart shows the literature screening process used in our report (Fig. 1). A total of 7681 potentially relevant studies were identified in the literature search, 7173 records were marked as ineligible by automation tools, and 216 of them were duplicates. Then, 292 studies were excluded after screening the titles and abstracts, and 23 studies were excluded after screening the full text. The remaining 14 eligible publications were collected for this meta-analysis.

### Studies and patient characteristics

A total of 14 [18–31] studies with 683 participants were included in the meta-analysis, of which 10 [18–21, 25–30] were RCTs, 3 [23, 24, 31] were controlled clinical studies, and 1 [22] was a retrospective study. The studies were performed in China [18, 21, 27, 30, 31], India [19, 20], Korea [24], Germany [25], Turkey [29], Czech Republic [22, 23], Iran [28], and Italy [26]. As for the stem cells applied, 2 used peripheral blood mononuclear cells (PBMNCs) [24, 29], 2 used bone marrow-derived mesenchymal stem cells (BMMSCs) [19, 27], 3 used bone marrow-derived mononuclear cells (BMMNCs) [23, 25, 27], 1 used bone marrow-enriched tissue repair cells (BMTRCs) [25], 1 used CD133^+^ cells [31], 1 used micro-fragmented adipose tissue [26]. 1 used human processed lipoaspirate cells (HPLAC) [24], 1 used bone marrow-derived cells (BMDC) [20], 1 used peripheral blood progenitor cells (PBPCs) [23], 1 used BMMNCs + PBPCs [22], and 2 used human umbilical cord mesenchymal stem cells (HUCMSCs) [21, 30]. The follow-up duration period varied from 2 to 18 months. Characteristics of included studies are presented in Table 1.

**Table 1:** Characteristics of the included studies

Author, year	Country	Study design	Mean age (treatment group, years)	Participant cases (treatment group/control)	Cell type	Amount of cells	Method	Control	Follow-up (month)
Huang, 2005	China	Rct	71	14/14	PBMNCs	3*10^9^/leg	i.m	PGE1	3
Dash, 2009	India	Rct		3/3	BMMSCs	10^6^/cm^2^	i.m	None	3
Han, 2010	Korea	Rct	67	26/26	PLAS	> 4 × 10^6^/ulcer	Ad.us.ext	None	2
Lu, 2011	China	Rct	65	41/41	BMMNCs	9.6*10^8^/ leg	i.m	NS	6
					BMMSCs	9.3*10^8^/leg	i.m	NS	6
Jain, 2011	India	Rct	54	23/24	BMDC	5 ml/leg		Peripheral blood	3
Kirana, 2012	Germany	Rct	69	12/6	BMMNCs	3*10^8^/leg	i.m. or i.a	None	11
				20/6	BMTRCs	8*10^7^/leg	i.m. or i.a	None	11
Ozturk, 2012	Turkey	Rct	71	20/20	PBMNCs	2*10^7^/leg	i.m	Conservative treatment	3
Dubsky, 2013	Czech Republic	Cct	61	17/22	BMMNCs	2.2*10^9^/leg	i.m	Conservative treatment	6
			63	11/22	PBPCs	2.4*10^10^/leg	i.m	Conservative treatment	6
Mohammadzadeh, 2013	Iran	Rct	64	7/14	PBMSCs	9–12*10^8^/leg	i.m	Sterile PBS	3
He, 2013	China	Rct	63	50/50	HUCMSCs	5.8–8.2*10^7^/ leg	i.m	Conservative treatment	3
DUBSKÝ	Czech Republic	Retrospective	63	31/23	BMMNCs + PBPCs	None	i.m	Conservative treatment	12
Qin, 2016	China	Rct	75	28/25	HUCMSCs	4.8–8.6*10^7^/leg	i.m	Conservative treatment	3
Zhang, 2016	China	Cct	71	27/26	CD133^+^ cells	≥ 1 × 10^7^/leg	i.a	NS	18
Leone, 2019	Italy	Rct	69	55/55	MFAT	10-30 ml/ leg	i.r.w	None	6

*Rct* Random controlled trial, *CCT* Controlled clinical trial, *PBMNCs* Peripheral blood-derived mononuclear cells, *BMMSCs* Bone marrow-derived mesenchymal stem cells, *PLAS* Human processed lipoaspirate cells, *BMMNCs* Bone marrow-derived mononuclear cells, *BMDC* Bone marrow-derived cells, *BMTRCs* Bone marrow-enriched tissue repair cells, *PBPCs* Peripheral blood progenitor cells, *PBMSCs* Peripheral blood mesenchymal stem cells, *HUCMSCs* Human umbilical cord mesenchymal stem cells, *MFAT* Micro-fragmented adipose tissue, *i.m*. Intramuscular injection, *i.a*. Intra-arterial injection, *i.r.w*. Injected radially into the wound, Ad.us.ext, ad usum externum (for external use), *NS* Normal saline, *PBS* Phosphate-buffered saline

### Quality of evidence

**Fig. 2:**
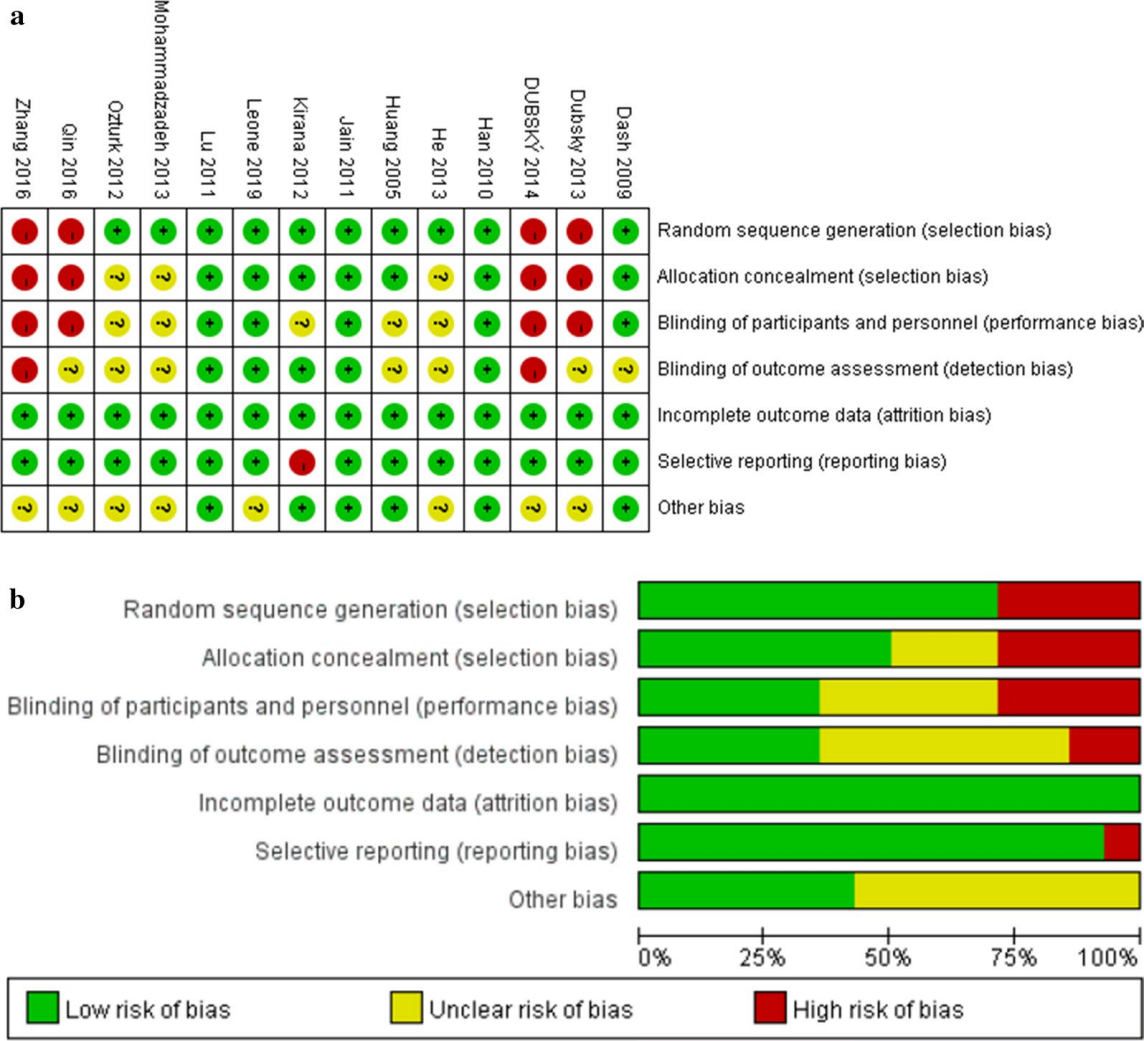
**a**: Risk of bias graph. **b**: Summary of study risk bias analysis

The risk of bias assessment of included studies using the Cochrane assessment tool is shown in Fig. 2a, b. The overall quality of included studies was moderate, and the quality of included studies varied from low to high. The included studies included 10 RCTs of relatively high quality. However, a total of 7 RCTs reported detailed methods for sequence generation and 4 RCTs did not mention specific methods for using blind methods for participants and implementers, which were therefore defined as uncertain risk. Three clinically controlled studies and 1 retrospective study were of low quality.

### Ulcer and wound healing rate

**Fig. 3:**
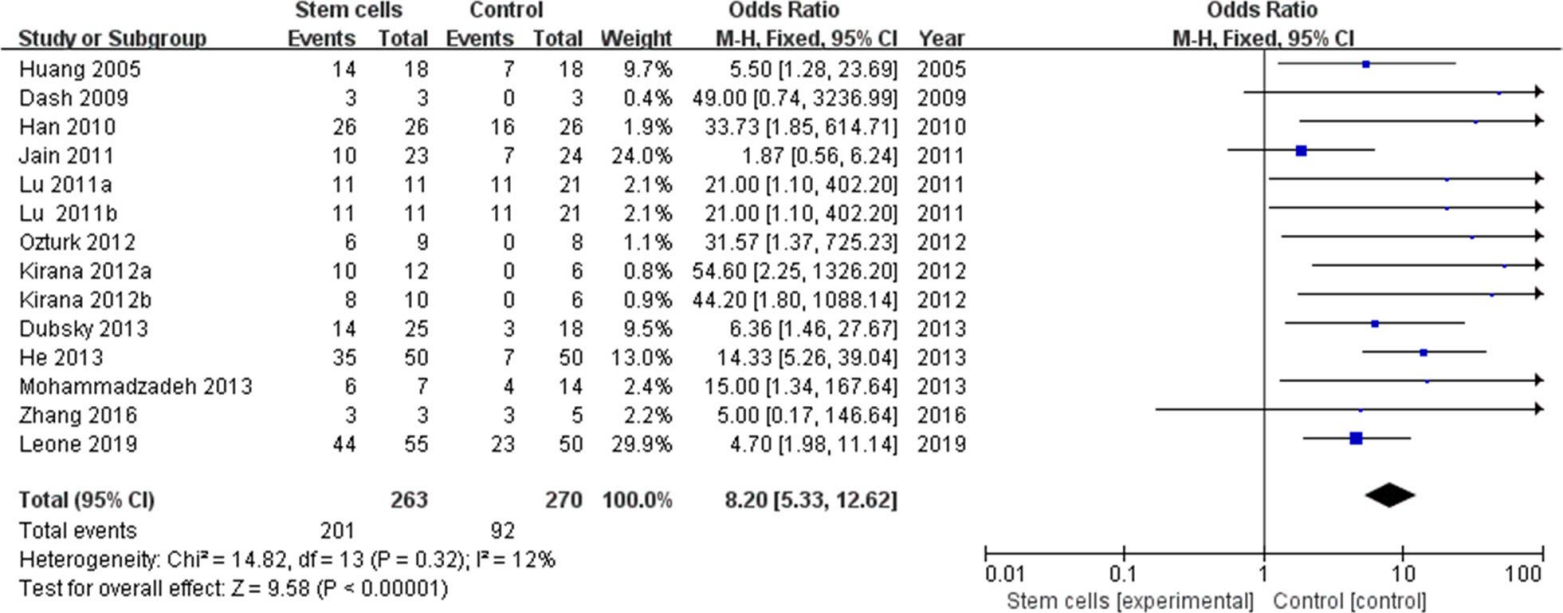
Forest plot showing the effect of stem cell therapy on ulcer or wound healing rate

Among the 14 included studies, 12 trials [18–21, 23–29, 31] reported detailed ulcer or wound healing rates. The meta-analysis showed that the healing rate of ulcers or wounds in the cell-treated group was higher than in the control group (201/263 vs 92/270 OR 8.20, 95% CI 5.33 to 12.62, *I*^2^ = 12%) (Fig. 3).

### Amputation rate

**Fig. 4:**
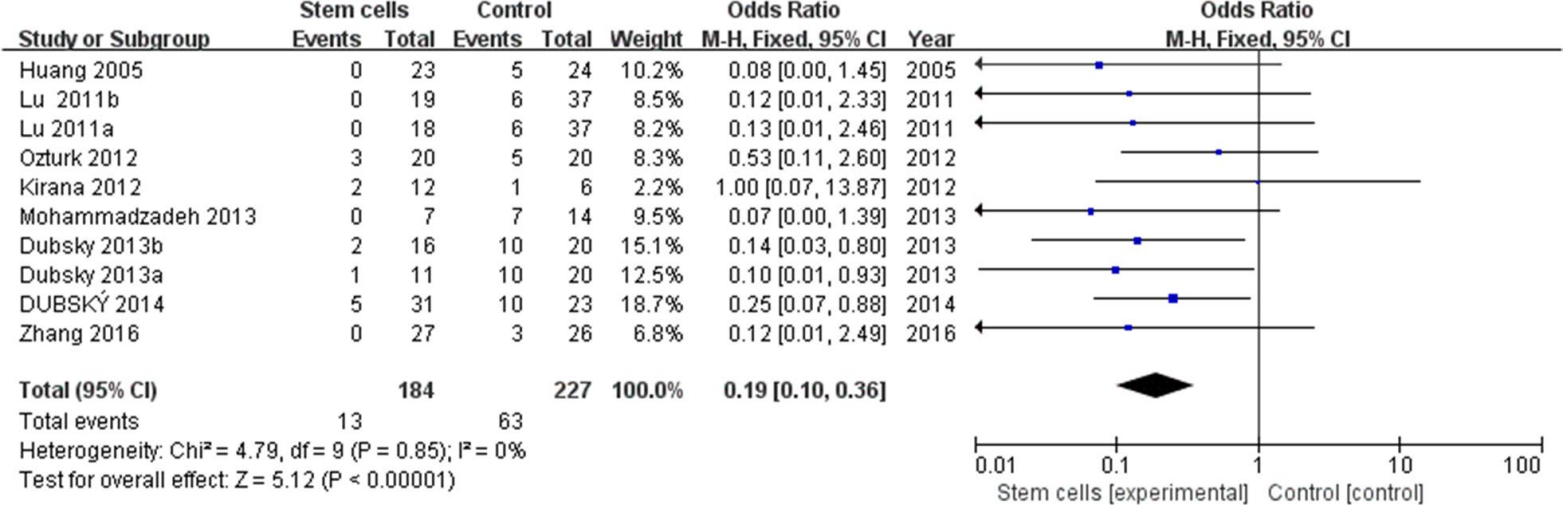
Forest plot showing the effect of stem cell therapy on amputation rate

Eight trials [18, 22, 23, 25, 27–29, 31] reported detailed amputation rates. The meta-analysis showed that the rate of amputation in the stem cell treatment group was significantly lower than in the control group (13/184 vs 63/227 OR 0.19, 95% CI 0.10 to 0.36, *I*^2^ = 0%) (Fig. 4).

### New vessels

**Fig. 5:**
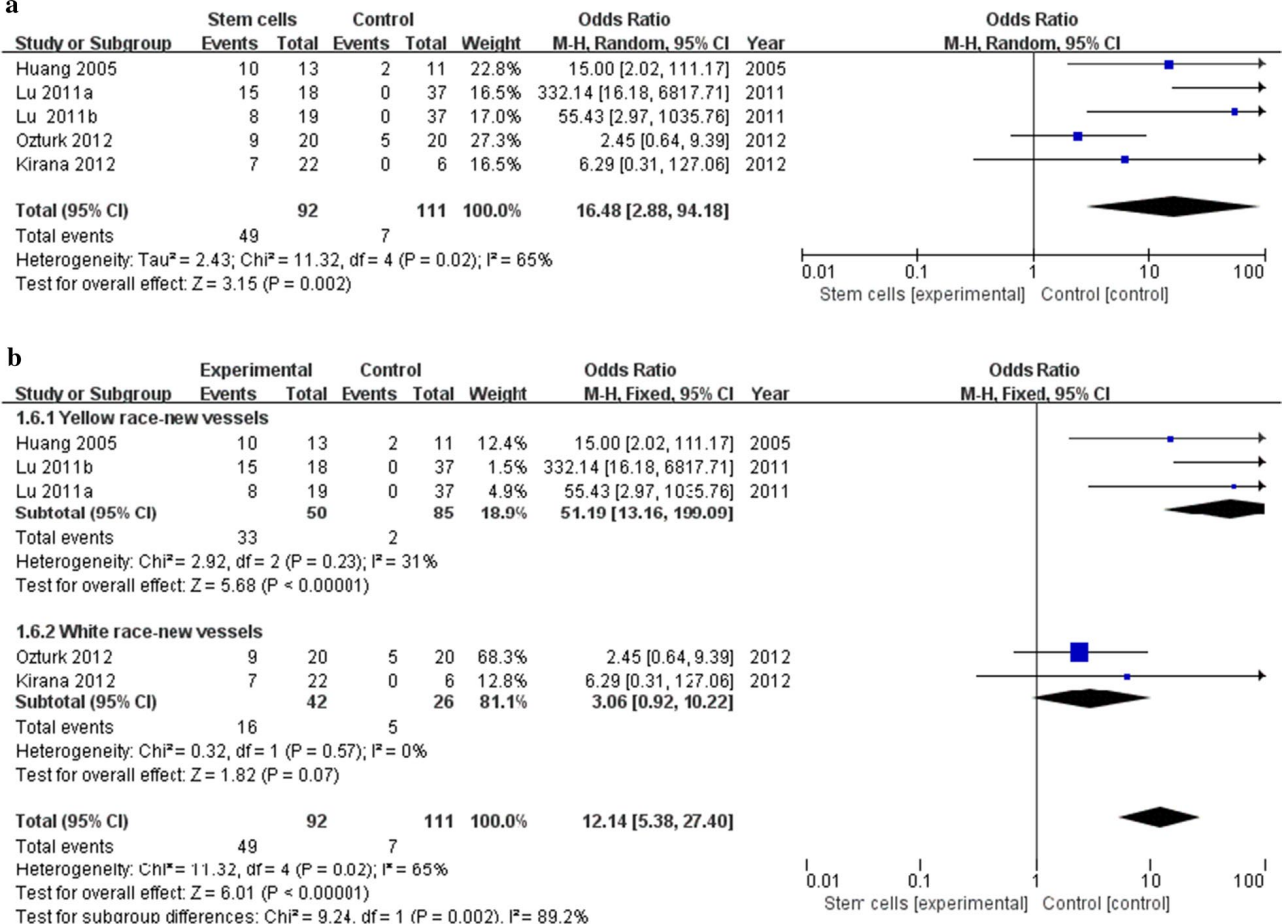
**a**: Forest plot showing the effect of stem cell therapy on new vessels. **b**: Subgroup analysis showed a forest plot of the effects of stem cell therapy on new vessels

Four studies [18, 25, 27, 29] reported detailed new angiogenesis rates. The meta-analysis showed that the rate of new angiogenesis was significantly higher in the stem cell treatment group than in the control group (49/92 vs 7/111 OR 16.48, 95% CI 2.88 to 94.18, *I*^2^ = 65%) (Fig. 5a). Due to its high heterogeneity of *I*^2^ = 65%, we performed subgroup analysis according to ethnic skin color. Meta-analyses of 2 studies [18, 27] in the yellow race showed that the rate of new angiogenesis in the stem cell treatment group was higher than that in the control group (33/50 vs 2/85 OR 51.19, 95% CI 13.16 to 199.09, *I*^2^ = 31%); Meta-analyses of 2 studies [25, 29] in the white race showed that the rate of new angiogenesis in the stem cell treatment group was higher than that in the control group (16/42 vs 7/111 OR 3.06, 95% CI 0.92 to 10.22, *I*^2^ = 0%)(Fig. 5b).

### ABI

**Fig. 6:**
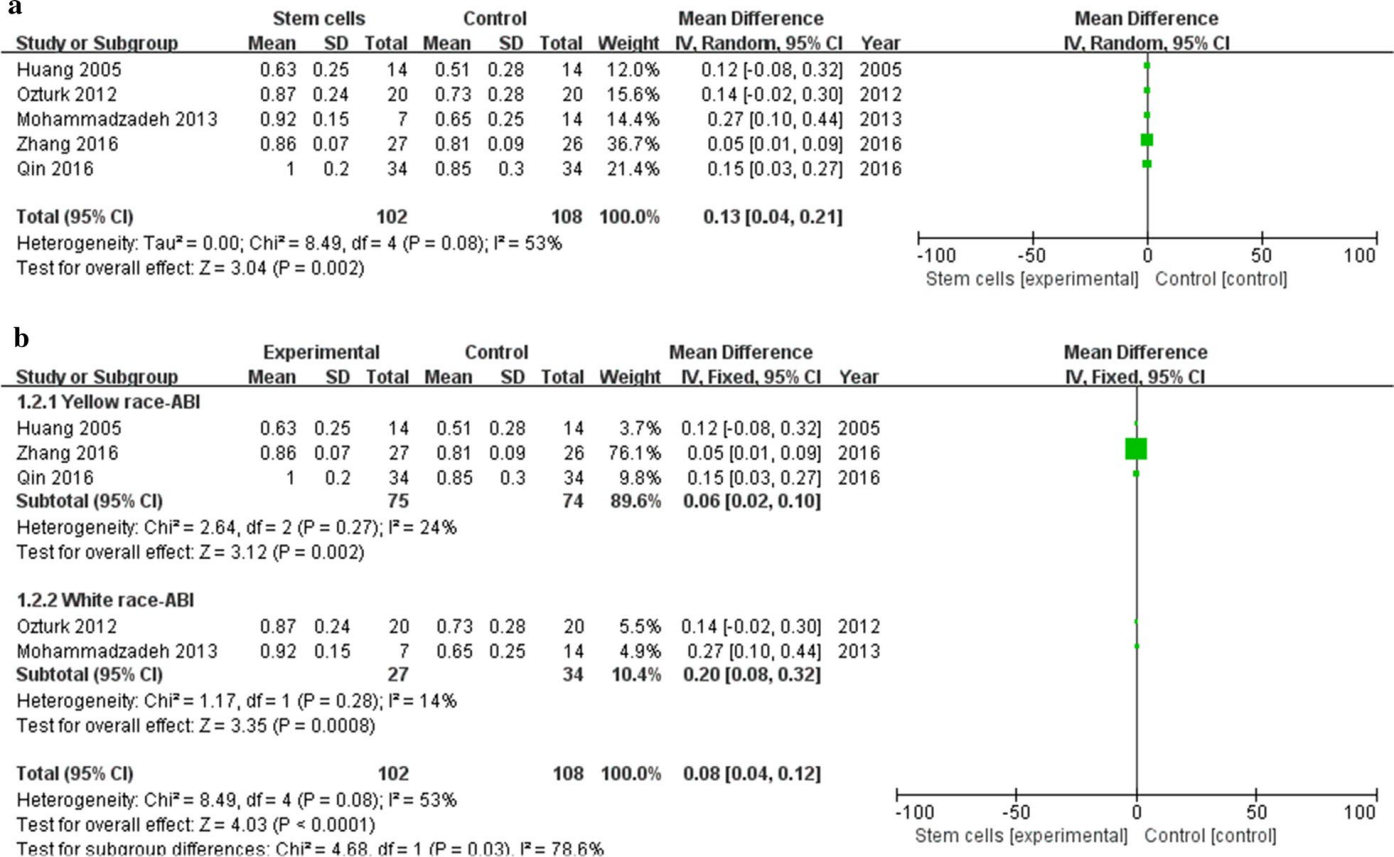
**a**: Forest plot showing the effect of stem cell therapy on ABI. **b**: Subgroup analysis showed a forest plot of the effects of stem cell therapy on ABI

Five studies [18, 28–31] reported detailed ABI. Meta-analysis shows higher ABI in cell therapy group than in control group (MD 0.13, 95% CI 0.04 to 0.08, *I*^2^ = 53%) (Fig. 6a). We performed subgroup analysis according to ethnic skin color. Meta-analyses of 3 studies [18, 30, 31] in the yellow race showed that the ABI in the stem cell treatment group was higher than that in the control group (MD 0.06, 95% CI 0.02 to 0.10, *I*^2^ = 24%); meta-analyses of 2 studies [28, 29] in the white race showed that the ABI in the stem cell treatment group was higher than that in the control group (MD 0.08, 95% CI 0.04 to 0.12, *I*^2^ = 14%) (Fig. 6b).

### TcPO_2_

**Fig. 7:**
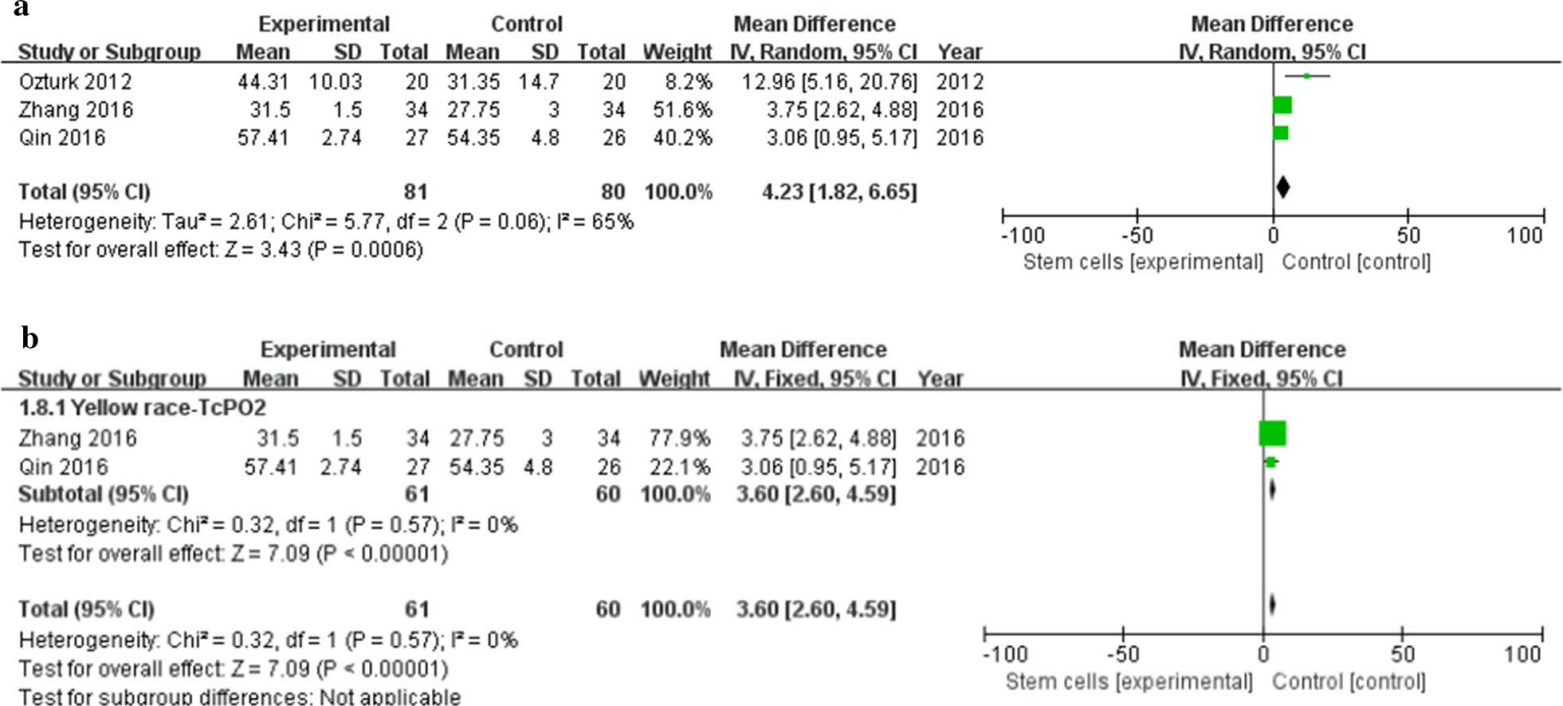
**a** Forest plot showing the effect of stem cell therapy on TcPO2. **b**: Subgroup analysis showed a forest plot of the effects of stem cell therapy on TcPO_2_

Three studies [29–31] reported detailed TcPO2. Meta-analysis shows that TcPO_2_ in the stem cell treatment group is higher than in the control group (MD 4.23, 95% CI 1.82 to 6.65, *I*^2^ = 65%) (Fig. 7a). Subgroup analysis showed that a meta-analysis of 2 studies [30, 31] in the yellow race showed that the TcPO_2_ of the stem cell treatment group was higher than that of the control group (MD 3.60, 95% CI 2.60 to 4.59, *I*^2^ = 0%) (Fig. 7b).

### Pain-free walking distance

**Fig. 8:**
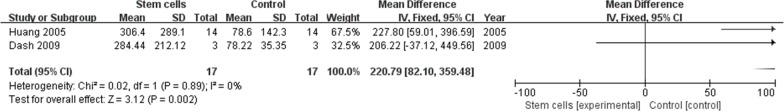
Forest plot showing the effect of stem cell therapy on Pain-free walking distance

Only 2 studies [18, 19] reported detailed pain-free walking distances. Meta-analysis shows that the pain-free walking distance in the stem cell treatment group is higher than that in the control group (MD 220.79, 95% CI 82.10 to 359.48, *I*^2^ = 0%) (Fig. 8).

### Rest pain score

**Fig. 9:**
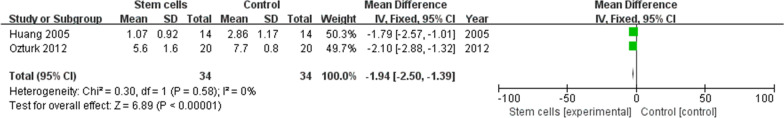
Forest plot showing the effect of stem cell therapy on rest pain score

Only 2 studies reported detailed rest pain scores. The meta-analysis showed that the rest pain score of the stem cell treatment group was lower than that of the control group (MD − 1.94, 95% CI − 2.50 to − 1.39, *I*^2^ = 0%) (Fig. 9).

### Sensitivity analyses

Results of sensitivity analyses are shown in Additional file 1: Figure S1, Additional file 2: Figure S2, and Additional file 3: Figure S3. The results of new vessels, ABI, and TcPO2 were highly heterogeneous, and the results were consistent with the original analysis after excluding the studies that caused the high heterogeneity.

### Adverse events

Adverse events were reported in only 5 studies [22, 23, 26, 27, 30] and were mainly pain in the recipient area and leg edema after stem cell transplantation.

### Publication bias

**Fig. 10:**
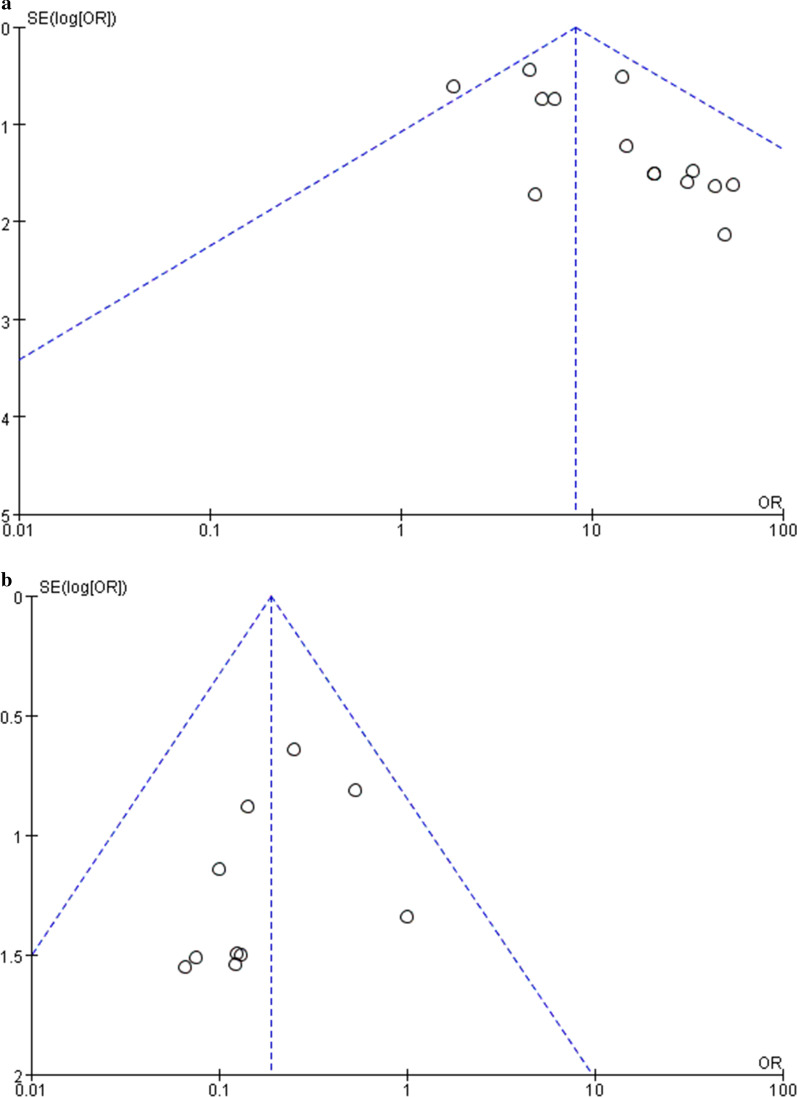
**a**, **b**: Publication bias in relation to ulcer or wound healing rate (10a) and amputation rate (10b)

Publication bias was qualitatively examined using funnel plots. The funnel plots of the effect of stem cell therapy on lower extremity ulcers or wound healing and the effect of amputation were nearly symmetrical on visual inspection, indicating no clear evidence of publication bias, as shown in Fig. 10 a and b.

## Discussion

According to the IDF report, the number of diabetes patients worldwide increased by 114.6 million from 2016 to 2021 and continues to rise due to current high-fat, high-sugar diets, sedentary lifestyles, and reduced physical activity [1, 14]. Diabetic foot is the most common chronic complication of diabetes. Diabetic foot is the most common chronic complication of diabetes. Its pathological manifestations are arteriosclerosis, occlusion, and neuropathy in the lower extremities, which leads to local accidental injury of the skin tissue of the foot or postoperative wounds that are difficult to heal and gradually form ulcers, resulting in amputation or even death [8, 32]. Diabetic foot not only reduces the quality of life, but also creates a huge economic burden, including direct and indirect costs of DF care and treatment. The traditional methods of treating diabetic foot are mainly systematic medical treatment, lower extremity surgical blood flow reconstruction, and wound debridement care [8, 12]. Drugs cannot fundamentally solve arterial stenosis, occlusion, and ischemia. Many small arterial lesions and the lack of distal arterial outflow tract, accompanied by cardiovascular and cerebrovascular diseases, are great challenges in surgery [9, 33]. Stem cells, platelet-rich plasma (PRP), and biomaterials show great potential in wound healing [34–37]. Angelis [35] et al. achieved good results in the treatment of chronic wounds caused by diabetes and vascular diseases by using the biological scaffolds made by platelet-rich plasma and hyaluronic acid, which not only accelerates the wound healing, but also reduces the pain of patients. Nicoli [38] et al. used PRP gel and Hyalomatrix PA to promote angiogenesis and stimulate wound dermal regeneration for wound healing after wide excision of severe Hidradenitis suppurativa. Porcine dermal matrix, double-layer dermal substitute, dermal regeneration template, and other biological materials have achieved good results in tissue reconstruction and repair in clinical practice [36, 39–42].

Stem cells are promising as a new treatment for diabetic foot. At present, the main types of stem cells and somatic cells used in the clinic are mesenchymal stem cells and mononuclear cells [11]. MSCs can be derived from umbilical cord, bone marrow, hair follicles, adipose tissue, gums, dental pulp, and menstrual blood, which are mainly used to treat diabetic foot by promoting angiogenesis, prolonging the reduction in the inflammatory period, and enhancing the content of type I collagen [12, 19, 21, 26, 27, 30, 43, 44]. Adipose-derived stem cells and adipose stem cells-rich stromal vascular fractions play an important role in chronic wound repair and regeneration after tissue defects [34, 45, 46]. Mononuclear cells are derived from bone marrow and peripheral blood, which have progenitor and stem cell characteristics, not require in vitro expansion. Mononuclear cells treat diabetic foot by promoting angiogenesis, reducing inflammation, enhancing re-epithelialization, and increasing collagen deposition [11, 18, 23, 27–29, 31]. Although somatic cells and stem cells are effective in the treatment of diabetic foot and there is some overlap in mechanism, it is still not clear which type of cells has a better therapeutic effect, and some high-quality clinical studies are still needed to explore.

Guo [14] et al. first conducted a meta-analysis of autologous stem cells in the treatment of diabetic foot ulcers in 2017. The results showed that compared with the control group, stem cells had better efficacy in the treatment of diabetic foot ulcers. However, only 4 trials were included, resulting in a low degree of confidence in the results. The meta-analysis reported by Shu [16] et al. in 2018 analyzed the efficacy of autologous stem cells in the treatment of diabetic foot ulcers, ABI and TcPO2; a total of 7 trials were included. The meta-analysis reported by Dai [13] et al. in 2020 included a total of 8 trials, which not only analyzed the efficacy of autologous stem cells in the treatment of diabetic foot ulcers, but also analyzed the amputation rate. The results showed that the amputation rate decreased after stem cell therapy. Fewer studies were included in these meta-analyses, and perhaps due to time reasons, fewer studies have been published on stem cells for the treatment of diabetic foot ulcers. The outcome indicators of DF treatment include not only the ulcer healing rate and amputation rate, but also the improvement in lower extremity ischemia and whether the patient's quality of life is improved after treatment.

This meta-analysis included 14 studies involving 683 patients with DF, which aimed to compare the efficacy of stem cell-based therapy with traditional treatments. After combining the study results, we found that after stem cell treatment, the ulcer or wound healing rate, the number of new vessels in the lower extremity, TcPO2, ABI, and pain-free walking distance increased significantly, while the amputation rate and rest pain score decreased significantly. This proves that the patient's physical condition and quality of life have been significantly improved after stem cell therapy. No significant change was observed with regard to the outcomes investigated, with the heterogeneity in the new vessels, ABI, and TcPO2 group that was decreased following the ethnic subgroup analysis. This implies that the therapeutic effect of stem cells is consistent in different races.

Although the sample size of our meta-analysis is large and the analysis indicators are comprehensive, there are still some limitations. (1) The included studies did not distinguish stem cells from other types of somatic cells. (2) Some of the included studies were of low quality, 4 were non-randomized controlled studies, and 3 RCTs did not describe specific methods for using blind methods for participants and implementers in detail, leading to combined conclusions. (3) Few studies are reporting the results of TcPO2, pain-free walking distance, and rest pain, which leads to lower reliability of the results after combined analysis.

## Conclusion

The meta-analysis of the current studies has shown that stem cells are significantly more effective than traditional methods in the treatment of diabetic foot and can improve the quality of life of patients after treatment. However, which cell type is most efficacious and the optimal parameters for cell therapy have not been determined. Future studies should conduct large-scale, randomized, double-blind, placebo-controlled, multicenter trials with high-quality long-term follow-up to demonstrate the most effective cell types and therapeutic parameters for the treatment of diabetic foot.

### Supplementary Information

**Additional file 1: Fig. S1. **Sensitivity analysis results for New vessels.

**Additional file 2: Fig. S2. **Sensitivity analysis results for ABI.

**Additional file 3: Fig. S3. **Sensitivity analysis results for TcPO2.

## Data Availability

DOI: https://doi.org/10.2337/diacare.28.9.2155, https://doi.org/10.1089=rej.2009.0872, https://doi.org/10.1111/j.1524-475X. 2010.00593.x, https://doi.org/10.1016/j.diabres.2010.12.010, https://doi.org/10.1111/j.1742-1241. 2011. 02,886.x, https://doi.org/10.1016/j.jdiacomp.2011.11.007, https://doi.org/10.1002/dmrr.2399, https://doi.org/10.1055/s-0032-1311646, https://doi.org/10.1016/j.jcyt.2014.08.010, https://doi.org/10.1055/s-0042-103684, https://doi.org/10.1155/2016/6925357, https://doi.org/10.1186/s13287-019-1328-4, PMID: 21,904,014, An article from CNKI: https://kns.cnki.net/kcms/detail/detail.aspx?dbcode=CJFD&dbname=CJFD2014&filename=ZMGY201404005&uniplatform=NZKPT&v=C9hVdG5mO8RB4NZ2y-GB_VetSFdofkdl1G98_Mkpn-f6l0luiA_hf1BMvzcLE1OE.
