# Supplementary figures and images for: Effectiveness and safety of stem cell therapy for diabetic foot: a meta-analysis update
Source: Stem Cell Res Ther. 2022 Aug 13;13:416. doi: 10.1186/s13287-022-03110-9 (PMC9375292; doi:10.1186/s13287-022-03110-9)

Figure S1


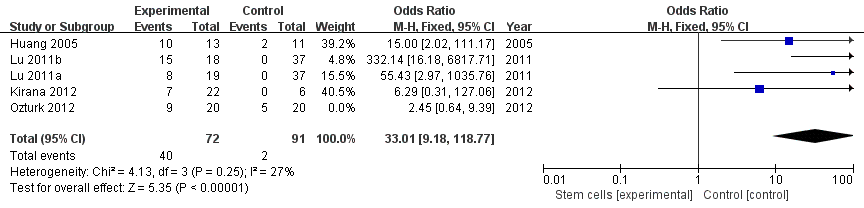

Supplement: Supplementary file 1 — Additional file 1: Fig. S1. Sensitivity analysis results for New vessels. [file 13287_2022_3110_MOESM1_ESM.docx]

Figure S2


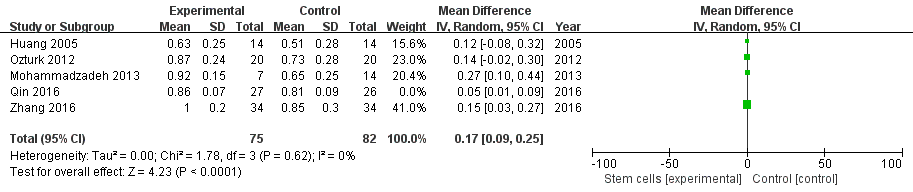

Supplement: Supplementary file 2 — Additional file 2: Fig. S2. Sensitivity analysis results for ABI. [file 13287_2022_3110_MOESM2_ESM.docx]

Figure S3


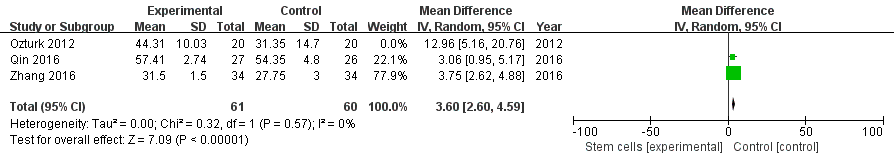

Supplement: Supplementary file 3 — Additional file 3: Fig. S3. Sensitivity analysis results for TcPO2. [file 13287_2022_3110_MOESM3_ESM.docx]
